# Complete Genome Sequence of Klebsiella quasipneumoniae Strain S05, a Fouling-Causing Bacterium Isolated from a Membrane Bioreactor

**DOI:** 10.1128/genomeA.00471-18

**Published:** 2018-06-07

**Authors:** Masaaki Kitajima, So Ishizaki, Jeonghwan Jang, Satoshi Ishii, Satoshi Okabe

**Affiliations:** a Division of Environmental Engineering, Faculty of Engineering, Hokkaido University, Sapporo, Hokkaido, Japan; b BioTechnology Institute, University of Minnesota, St. Paul, Minnesota, USA; c Department of Soil, Water and Climate, University of Minnesota, St. Paul, Minnesota, USA

## Abstract

We report here the complete genome sequence of Klebsiella quasipneumoniae strain S05, a bacterium capable of producing membrane fouling-causing soluble substances and capable of respiring on oxygen, nitrate, and an anodic electrode. The genomic information of strain S05 should help predict metabolic pathways associated with these unique biological properties of this bacterium.

## GENOME ANNOUNCEMENT

A membrane bioreactor (MBR) is an advanced wastewater treatment technology that achieves improved effluent water quality with less demand for space (1). However, application of this technology in full-scale plants has been largely limited by membrane fouling, which causes a drastic decrease in membrane flux associated with increased cost/energy requirements for operation (2, 3). Previously, we isolated bacterial strains responsible for membrane fouling, termed fouling-causing bacteria (FCB), from fouled membranes in a pilot-scale MBR fed with real municipal wastewater (4). One of the FCB, Klebsiella quasipneumoniae strain S05, was found to excrete soluble microbial products (SMPs) that cause severe membrane fouling (4, 5). Interestingly, this strain is also capable of respiring on various electron accepters, including solid-state anodic electrodes and oxygen and nitrate (S. Ishizaki, P. R. Islam, H. Miyake, Y. Narita, and S. Okabe, unpublished data). Complete genome sequencing was thus conducted to characterize the genomic features of strain S05, an exoelectrogenic FCB exhibiting these unique biological properties.

Strain S05 was grown in R2A medium overnight at 30°C, and genomic DNA was extracted using the PowerBiofilm DNA isolation kit (Mo Bio Laboratories). Sequencing libraries were prepared using the PacBio single-molecule real-time (SMRT) kit (Pacific Biosciences), and the genome was analyzed using the PacBio RS II platform (Pacific Biosciences). After quality filtering, a total of 94,140 reads were obtained with a mean read length of 14,540 bp. The resulting high-quality reads were assembled *de novo* by using the Hierarchical Genome Assembly Process 3 (HGAP3) in the SMRT Link portal (v 2.3.0). Average nucleotide identity (ANI) values were calculated using JSpecies (6).

The resulting assembly of strain S05 consisted of one circular chromosome with a size of 5,157,054 bp and a G+C content of 58.0%. Genome annotation was done using the NCBI Prokaryotic Genome Annotation Pipeline (7), which identified 4,783 protein coding sequences (CDS), 225 pseudogenes, 86 tRNAs, 25 rRNAs (8 rRNA operons), and 9 noncoding RNAs. The ANI values between the genomes of strain S05 and type strains of Klebsiella *quasipneumoniae* (subsp. *quasipneumoniae* strain 01A030^T^, GenBank accession number CCDF00000000) (8), Klebsiella pneumoniae (ATCC 13883^T^, GenBank accession number JOOW00000000) (9), and Klebsiella *variicola* (DSM 15968^T^, GenBank accession number CP010523) were 96.5%, 93.7%, and 93.4%, respectively. The ANI value with the type strain of *K. quasipneumoniae* was greater than the cutoff value for species discrimination (95% to 96%) (6, 10). An even higher ANI value of 99.1% was observed between strain S05 and the type strain of *K. quasipneumoniae* subsp. *similipneumoniae* (07A044^T^, GenBank accession number CBZR000000000) (8). These results suggest that strain S05 belongs to the species *K. quasipneumoniae*, although strain S05 was previously considered to be most closely related to K. pneumoniae, based on the 16S rRNA gene sequence similarity of 99.5% (5).

The complete genome information of strain S05 should help predict metabolic pathways associated with the synthesis of SMPs that are responsible for membrane fouling. With the genome data, we also expect to identify genes associated with its respiration.

### Accession number(s).

The complete genome sequence of K. quasipneumoniae strain S05 has been deposited into the NCBI database under the accession number CP024784.
